# Cell Fragmentation and Permeabilization by a 1 ns Pulse Driven Triple-Point Electrode

**DOI:** 10.1155/2018/4072983

**Published:** 2018-03-18

**Authors:** Enbo Yang, Joy Li, Michael Cho, Shu Xiao

**Affiliations:** ^1^Frank Reidy Research Center for Bioelectrics, Old Dominion University, Norfolk, VA, USA; ^2^Department of Biomedical Engineering, Cornell University, Ithaca, NY, USA; ^3^Department of Bioengineering, University of Texas, Arlington, TX, USA; ^4^Department of Electrical and Computer Engineering, Old Dominion University, Norfolk, VA, USA

## Abstract

Ultrashort electric pulses (ns-ps) are useful in gaining understanding as to how pulsed electric fields act upon biological cells, but the electric field intensity to induce biological responses is typically higher than longer pulses and therefore a high voltage ultrashort pulse generator is required. To deliver 1 ns pulses with sufficient electric field but at a relatively low voltage, we used a glass-encapsulated tungsten wire triple-point electrode (TPE) at the interface among glass, tungsten wire, and water when it is immersed in water. A high electric field (2 MV/cm) can be created when pulses are applied. However, such a high electric field was found to cause bubble emission and temperature rise in the water near the electrode. They can be attributed to Joule heating near the electrode. Adherent cells on a cover slip treated by the combination of these stimuli showed two major effects: (1) cells in a crater (<100 *μ*m from electrode) were fragmented and the debris was blown away. The principal mechanism for the damage is presumed to be shear forces due to bubble collapse; and (2) cells in the periphery of the crater were permeabilized, which was due to the combination of bubble movement and microstreaming as well as pulsed electric fields. These results show that ultrashort electric fields assisted by microbubbles can cause significant cell response and therefore a triple-point electrode is a useful ablation tool for applications that require submillimeter precision.

## 1. Introduction

Electric pulses in the range of several nanoseconds to picoseconds are an important electric stimulus for studying ultrafast biological processes initiated by electric fields. As the pulse is shorter than a cell membrane charging time, which is typically estimated to be 100 ns or longer, it can be hypothesized that the pulse penetrates the cell membrane and affects the cell organelles by circumventing the shielding from the membrane charging. Intracellular manipulation in principle is more likely than with longer pulses. Although this hypothesis has not been rigorously proven, there is evidence that shorter pulses are indeed more efficient to recruit calcium from intracellular stores [1]. However, as the pulse becomes shorter, the threshold to induce biological effects such as stimulation or electroporation becomes higher, as predicted by a typical strength duration curve [2]. A series of works conducted in the 1990s showed that subnanosecond pulses caused minor or no effects on heart rate and blood pressure in live animals [3, 4]. In the later work on gastrocnemius muscles isolated from frogs, it was shown that muscle excitation requires a much higher field than those used in the animal experiments [5]. In the in vitro experiments, subnanosecond pulses were used to study membrane permeabilization. Still, a large field is needed to cause trypan blue uptake. For a cell that is rich in voltage-gated channels, a single subnanosecond pulse can permeabilize the membrane, presumably through the voltage-gated channels rather than the lipid bilayer, but the field is still rather high (~200 kV/cm) [6–8]. When subnanosecond pulses are applied repetitively, temperature increase contributes to the cell killing in addition to the pulsed electric field [9] and so the electric field can be effectively lowered to 20 kV/cm. Temperature was found to sensitize the membrane so the permeabilization is more probable to the electric fields [10].

Generation of high electric fields usually relies on a high voltage (200 kV) subnanosecond pulse generator [11], which is generally bulky and not easy to use and therefore limits its applicability in most biology labs. Recently, the technology to generate moderate or low voltage (<10 kV) subnanosecond pulses has advanced and generators are commercially available. Such low voltages allow us to generate electric fields that can be as high as 200 kV/cm with two wires in parallel for small-diameter electrodes and a short electrode gap distance [8]. It is also possible to use the triple-point effect to enhance the electric field near the metallic electrode with a low dielectric constant sleeve. Such an electrode was reported in [12] as a pulsed electron avalanche knife (PEAK) for eye surgery. It was later studied as a component in a water trigatron to initiate water breakdown [13]. This electrode may allow us to generate a sufficient field with even lower voltage.

When high intensity pulses are delivered to cells with electrodes, not only are pulsed electric fields applied to cells, but also side products, such as pressure transient, are generated near electrodes. This pressure transient can be the result of electrostriction, in which water dipoles experience a sudden electric force for aligning with the electric field [14, 15]. A recent experiment showed that a pressure transient can be produced by nanosecond pulses [16]. In addition to pressure transient, microbubbles are also commonly observed near the electrodes [17, 18] and have been postulated as the precursor for water streamer formation as electronic processes such as water molecular ionization and dissociation can occur inside [19, 20]. Free radicals can also form and cause downstream electrochemical effects [21]. Furthermore, bubble collapse may also induce a water jet that results in a high speed shock, which further complicates the physical picture [22, 23]. In general, delivery of high intensity pulses involves a series of side effects although pulsed electric field is often perceived as the sole event.

In this paper, we report the use of a triple-point electrode (TPE) to deliver 1 ns pulses as a tool to study in vitro responses. The main rationale of using TPE is to amplify the electric field for low-voltage, ns-ps pulses and provide an easy-to-use approach of studying the biological responses. The TPE was installed on a microscope on top of a cover slip preseeded with cells and real-time images were taken while the pulses were delivered. As a high intensity electric field is needed for 1 ns pulses to act on cells, byproducts, including pressure waves and microbubbles, are expected to accompany the electric field. It is likely that the local temperature near the electrode will increase. We quantified each aspect through either measurement or computer simulation, aiming to clarify the role of electric field, bubbles, and temperature. The impact of these factors on cells was analyzed from bright-field images and fluorescence images of propidium iodide (PI) staining. We found that cells were fragmented after exposure to the combination of all stimuli (electric field, microbubbles, and temperature increase) due to the pulse application. The fragmentation was confined to a crater with a diameter of 100 *μ*m. It is a rather severe effect and was typically not observed in cells after exposure to nanosecond pulse electric fields using conventional needle electrodes. Electric field alone is not sufficient to cause this effect and mechanical force should be taken into account. A less severe effect observed was the membrane permeabilization for cells that were on the crater periphery, where the electric field is close to the electroporation threshold. These cells can be useful for further analysis to understand the impact of pulses on cell recovery and survival.

## 2. Materials and Methods

### 2.1. Exposure System

**Figure 1:**
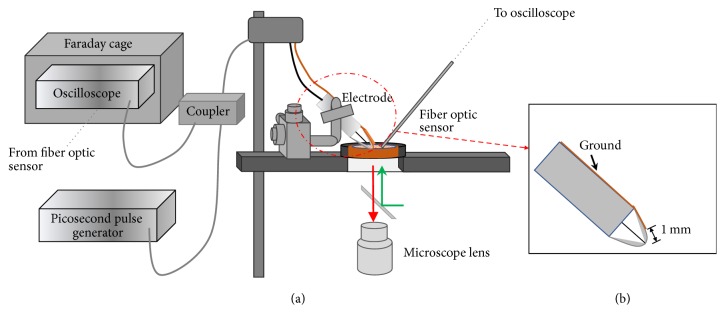
Delivery of 1 ns pulses to cells on a microscope stage through a glass-insulated triple-point electrode (TPE). (a) The setup. (b) The TPE electrode was brought close to cells on a cover slip by a micromanipulator.

Cell cover slips with precultured Chinese Hamster Ovarian (CHO) cells were placed in a microcuvette on a microscope (IX71, Olympus America, Waltham, MA). The TPE was mounted on a 3D movable stage, so the electrode could be maneuvered on top of the cover slip (Figure 1). The electrode was connected to the inner conductor of a coaxial cable through a lead. Another lead working as a ground return was connected to the outer conductor of the coaxial cable. In the process of selecting electrodes, several different electrodes were explored: (a) a bare tungsten needle electrode, where this electrode has a tip diameter of 5 *μ*m and its highest electric field is near its tip; (b) a tungsten needle electrode (5 *μ*m tip diameter) encapsulated with epoxy glue; this electrode tip is exposed to air, but the rest of the body is covered with glue; and (c) a tungsten wire electrode (50 *μ*m diameter) sealed in a glass tube. This electrode was fabricated by melting and pulling a glass pipette with the wire inside. Afterwards, the glass-encapsulated wire was polished until a smooth glass surface was formed with the wire exposed. Both b-type and c-type electrodes can be triple-point electrodes (TPE) once they are immersed in water, and the electric field can be enhanced near the triple-point locations. Each of these electrodes was supposed to produce the largest electric field near its tip and, as a result, microbubbles can be produced when pulses were applied to the electrode. For the a-type electrode, besides bubbles that were produced at the tip, a few bubbles were also generated on the tungsten rod surface away from the tip, presumably due to some invisible sharp points. For the b-type electrode, microbubbles were produced at the tip of the electrode, but some bubbles were also trapped in some microscale gap regions which the epoxy glue did not completely cover. For the c-type electrode, bubbles were emitted only from the tip and the bubble generation was found only at the tip of the electrode, which led us to choose it as our experimental electrode.

A picosecond-rise time pulse generator (FPG-5P, FID GmbH, Germany) supplied pulses to the TPE with a peak voltage of ~+4 kV. The pulses were continuously applied to TPE at 1 kHz for various durations. The pulses were measured by an inline capacitive sensor (VDC-1, Farr Research, NM), which measures the displacement current (i.e., the time derivative of the pulse) in the cable. The waveform on the oscilloscope (TDS7404, Tektronix, Beaverton, OR) was then integrated in order to obtain the voltage waveform. To measure the temperature change during pulsing, a fiber optic sensor (T1C-11000A, Neoptix Inc., Canada) was placed near the electrode using another micromanipulator stage. The fiber was connected to a computer via a controlling unit (RFX-04-1, Neoptix Inc., Canada).

### 2.2. Cell Culture and Propidium Iodide Staining

CHO cells were cultured in F-12 K medium with 10% FBS and 1% Penicillin-Streptomycin-Glutamine on a 12 mm in diameter cover slip and were kept in an incubator at 37°C and 5% CO_2_ until experimentation. In the experiments, the cover slip was placed in a microcuvette containing the same medium. A camera (DP80, Olympus America, Waltham, MA) with a fastest speed of 20 fps recorded the images during the pulsing. PI was used as a bioprobe to detect membrane integrity. 500 *μ*l of cell culture medium including PI with a final concentration of 5 *μ*g/ml was added to the microscope chamber and the coverslip was then put in the bottom of the chamber. Cells seeded in the coverslip were facing up towards the electrode.

## 3. Experiment Results

### 3.1. Electric Field Near the TPE

**Figure 2:**
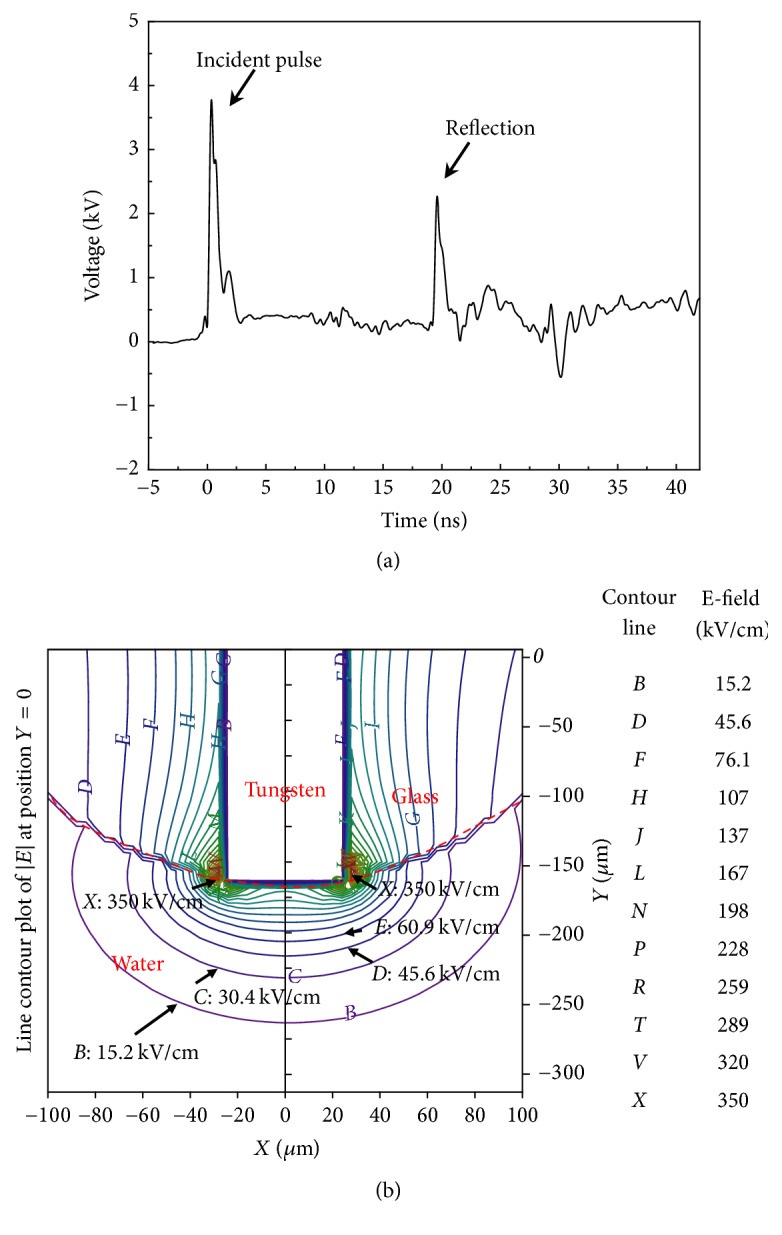
1 ns pulses delivered to the TPE. (a) The pulse was integrated on the oscilloscope from the time derivative signal (not shown) obtained by the capacitive probe. (b) Electric field distribution near the electrode.

The waveform of the pulses delivered to the TPE is shown in Figure 2(a). An incident pulse, which has a full width at half maximum (FWHM) of approximately 1 ns, was delivered to the electrode. As a result, a pulse was reflected due to the impedance mismatch between the TPE and the coaxial cable. These two pulses allowed us to calculate the approximate resistance of the TPE, as done in the practice of time-domain reflectometry. Since the incident pulse peak voltage is *V*_in_ = 3.75 kV and the return voltage is 2.2 kV, the reflection coefficient *R* is 0.58. The resistance of TPE is

(1) $$\begin{array}{c} R_{out}=Z_{line}\times \frac{1+R}{1-R}, \end{array}$$

where *Z*_line_ = 50 Ω. *R*_out_ is calculated as 188 Ω. The actual voltage at the TPE should be

(2) $$\begin{array}{c} V_{TPE}=V_{in}\times \frac{2R_{out}}{R_{out}+Z_{line}}, \end{array}$$

which is 5.9 kV. Knowing the voltage allowed us to calculate the electric field near the electrode (Field Precision, Albuquerque, NM). The electric field distribution near the TPE for a given voltage of 1 kV is shown in Figure 2(b). The highest electric field is near the interface of water, glass, and tungsten wire. In the experiment, the highest electric field was calculated as 2.1 MV/cm for the voltage at the electrode tip (5.9 kV), but the highest field is only 0.63 MV/cm for the electrode without glass encapsulation. At 25 *μ*m on the electrode axis from the electrode tip, the electric field is 0.63 MV/cm for the TPE and is only 0.279 MV/cm for the electrode without glass encapsulation. It is thus beneficial to use TPE to enhance the local electric field. The decrease of electric field, however, is very fast over distance. Even at a distance of 100 *μ*m, the electric field already decreases to 90 kV/cm.

### 3.2. Bubble Generation

**Figure 3:**
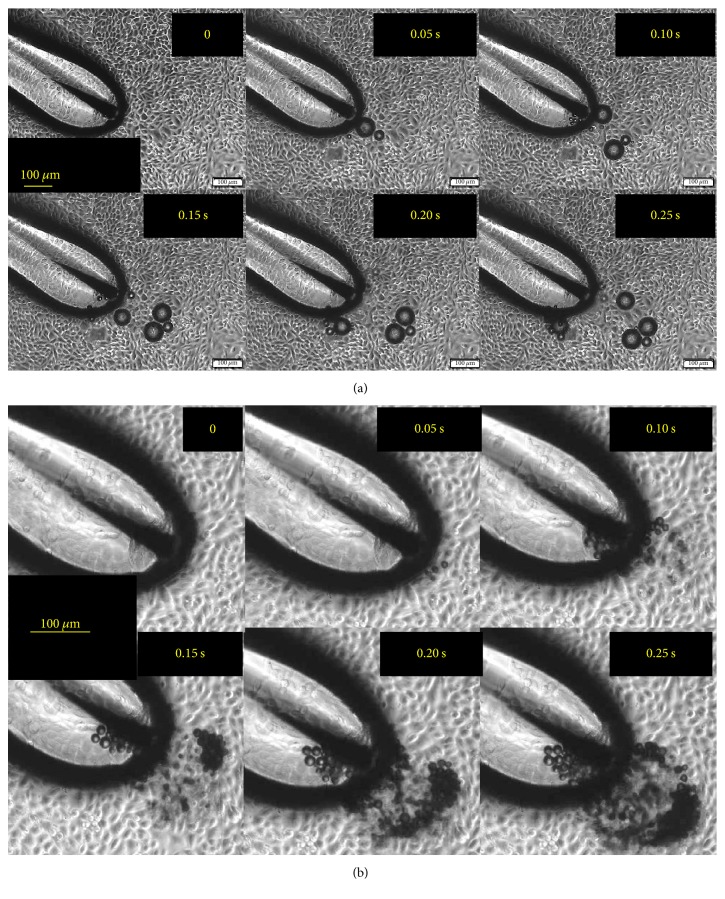
Bubbles generated in separate experiments: (a) the bubbles with approximately the same diameter of ~80 *μ*m were generated in 0.25 s when the pulses were applied and (b) the bubbles that have various sizes (mostly <20 *μ*m) were generated in the form of clusters.

Microbubbles were generated as soon as the pulses were applied to the electrode, but the sizes were not uniform. The diameter of the largest bubble never exceeded 100 *μ*m, whereas the smallest bubble size was hard to determine due to the limitation of the spatial resolution of the camera. A rough estimation from Figure 3 suggests that it can be much less than 20 *μ*m. Two special cases are shown in Figure 3, where (1) mainly large bubbles (~80 *μ*m in diameter) and (2) only bubble clusters consisting of small bubbles (<20 *μ*m) were produced. Most of the time, a mixture of large bubbles and bubble clusters were observed near the electrode. For a large bubble to grow, it took no longer than 0.05 s, shown in Figure 3(a). Almost in every 0.05 s, a large bubble was produced. But such “linear” bubble growth pattern was not seen in Figure 3(b). Rather, the small bubbles grew in an “explosive” manner from 0.15 to 0.25 s, showing the complexity of the bubble growth dynamics.

### 3.3. Temperature Change

**Figure 4:**
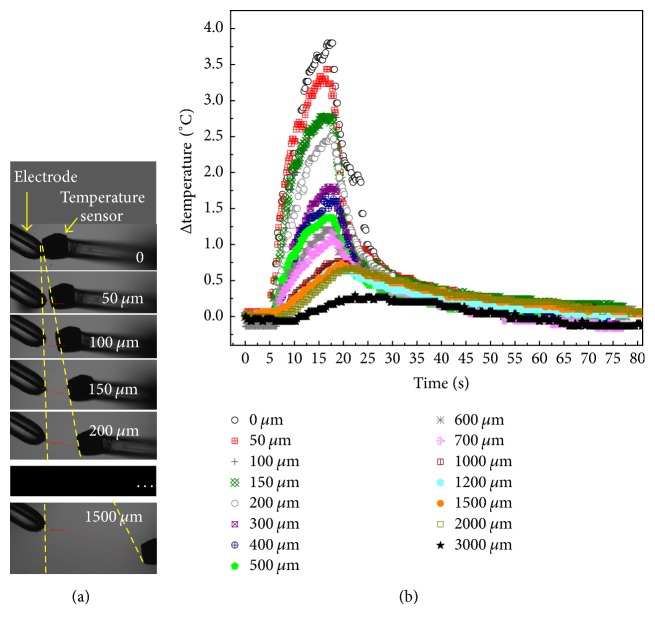
Temperature was measured with a fiber optic sensor. (a) The relative position of the sensor to the electrode; (b) the temperature change over time at different locations.

To measure the temperature change near the electrode, an optic fiber sensor was placed at varying distances (0 to 3000 *μ*m) from the electrode (Figure 4(a)). Here, the cover slip was not used, but both the electrode and fiber sensor were in the cell culture medium. For each distance, the pulses were applied for 15 s. At all distances, temperature continued increasing throughout the pulsing and dropped as soon as pulses were stopped (Figure 4(b)). At a distance of 3000 *μ*m, the increase in temperature was only 0.6°C. After pulsing, all temperatures dropped back to the baseline in about 1 minute. Clearly, the bulk of the fluid was at the ambient temperature.

### 3.4. Cell Response

**Figure 5:**
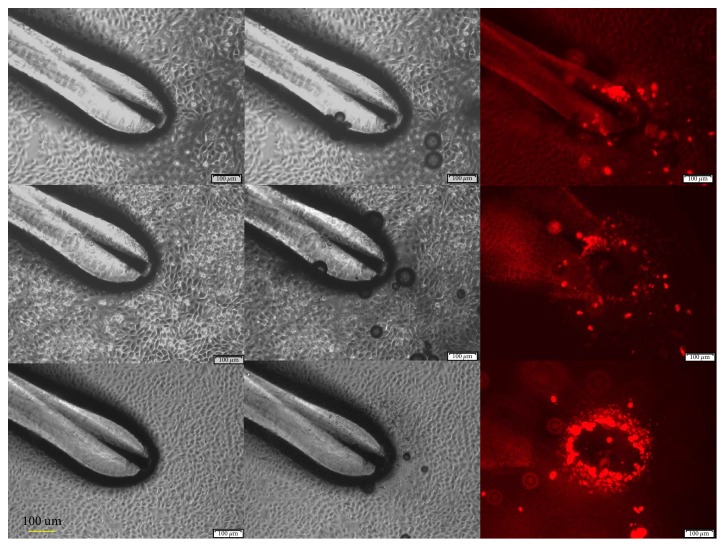
CHO cells were treated for 1 s, 2 s, and 4 s by a TPE energized with 1 ns pulses. The bright-field images and PI uptake are shown.

After exposure of 1 ns pulses for 1 s, 2 s, or 4 s, the resulting cell response is shown in Figure 5. After 1 s exposure, there were only a few cells that took up PI and not much change was observed in cell morpohology. After 2 s exposure, the pulses again did not produce much change in cell morphology but caused more PI uptake in cells. However, after the 4 s exposure, cell PI uptake became obvious and was observed for cells surrounding the TPE tip. These cells were distributed in a circle with a radius of approximately 100 *μ*m, forming a crater. Inside the crater, no PI uptake was observed. A careful examination of the images (4 s) indicated that the cells inside the circle were mostly wiped out. In front of the TPE, much small debris was observed, which was cell fragments resulting from broken cells.

**Figure 6:**
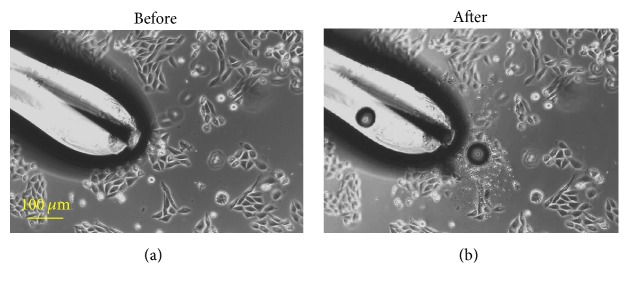
Cell fragmentation near the tip of TPE before and after applying 1 ns pulses: (a) before the exposure and (b) after the 1 s exposure.

A closer view of the damaged cells was obtained for pulsing low-confluence cells (30%) (Figure 6) for 1 s. Several cells that were close to the TPE tip were chosen for observation. After exposure, the cells were broken into fragments. This damage was rather local and confined within a distance of 100 *μ*m from the electrode tip. Outside the region, cells were intact.

**Figure 7:**
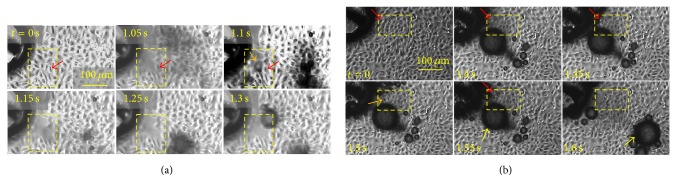
Time-resolved images for cell detachment near the tip of TPE during the application of 1 ns pulses. (a) A bubble (*t* = 1.1 s, indicated by the orange arrow, ~30 *μ*m diameter) wiped out a cell (indicated by the red arrow), which is presumably caused by its collapse. In the meantime, other adjacent cells remained attached. (b) In a different experiment, a cell (indicated by the red arrow *t* = 0, 1.4 s, 1.45 s, and 1.55 s, denoted as cell C) was wiped out from the cover slip and a bubble (indicated by the yellow arrow at *t* = 1.55 s and 1.6 s, denoted as bubble A, ~100 *μ*m diameter) was pushed away, which were caused by the same event, presumably a bubble collapse in the proximity of cell C. The cell indicated by an orange arrow was also wiped out in the same time.

Besides cell fragmentation, individual cells near the TPE were wiped out from the cover slip, which could take place in 0.05 s, as shown in the temporally resolved images (Figure 7).  Figure 7(a) shows that a bubble burst caused the cell removal. A cell pointed out by the red arrow managed to stay attached to the cover slip for 1.1 s after pulse application despite the attacks of many bubbles and pulsed electric fields. However, it was removed when a bubble showed up at *t* = 1.1 s (indicated by the orange arrow) to its close proximity and collapsed prior to 1.15 s. Meanwhile, most of other adjacent cells still attached to the cover slip. This removal caused by the bubble burst is rather local and only confined to a single cell. In Figure 7(b), two cells (one indicated by a red arrow inside the yellow rectangle at *t* = 0 s, 1.4 s, 1.45 s, and 1.55 s and the other indicated by an orange arrow at 1.5 s, Figure 7(a)) vanished between 1.55 s and 1.6 s. Simultaneously, a large bubble (~100 *μ*m diameter, indicated by the yellow arrow) near the cell was pushed away by 170 *μ*m at an average speed of 0.34 cm/s. Notice that the bubble stayed almost at the same location from 1.4 s to 1.55 s before being translated to its new location at 1.6 s. Apparently, the bubble displacement and cell removal were caused by the same event, which was most likely a bubble collapse as observed in Figure 7(a).

## 4. Discussions

The damage to adherent cells (CHO cell) after exposure to high intensity 1 ns pulsed electric fields delivered by a glass-encapsulated TPE includes (1) cell fragmentation that occurred in the region next to the TPE tip with the highest field, where the cells were fragmented and the cell fragments were blown away, forming a cell-free crater, and (2) cell permeabilization was found on those cells that surround the crater. The size of the crater was approximately 100 *μ*m in diameter; thus, the damage was rather local.

**Figure 8:**
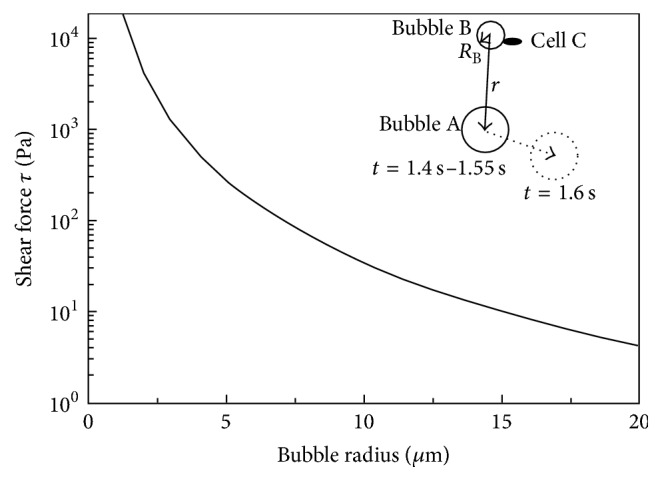
The shear force on a cell (cell C) calculated from a hypothetical, adjacent bubble collapse (bubble B). The fluid velocity associated from bubble B was extrapolated from bubble A's average velocity from 1.55 s to 1.6 s, which was obtained from Figure 7(b).

The formation of the crater and cell fragmentation are a rather severe effect and are not seen in cells exposed to low intensity electric fields, where cell shapes are preserved. When intense electric pulses are applied to the electrode and consequently to the cells on the cover slip, the action is very complex and multifaceted, involving electric field, bubble emission, pressure waves, and an increase of temperature. To elucidate the role of each is difficult with our current experimental setup. Although intense electric fields can cause cell swelling and blebbing, direct cell fragmentation has not been reported. Instead, it is more likely to be caused by microbubble collapse as Figure 7 clearly shows that bubble is responsible for the cell detachment, a milder effect than cell fragmentation. However, the bubbles can be more energetic in the crater region as they become closer to the electrodes. A generally accepted theory of bubbles imposing damage on adherent cells is that when a bubble collapses asymmetrically on the cover slip surface, a high speed microjet forms towards the surface, which is violent enough to rupture a cell membrane. The vorticity along the jet can cause secondary, smaller bubbles to form and rotate in an asymmetrical manner. The associated shear stress can further permeabilize cell membrane or detach the cell. In our setup, we could not capture such a picture due to the hardware limitation, but a rough estimation of a possible shear from a bubble collapse can be made based on Figure 7(b). The bubble (indicated by the yellow arrow at *t* = 1.55 s and 1.66 s in Figure 7(b), denoted as bubble A) was translated by 170 *μ*m within 0.05 s at an average speed of 0.34 cm/s from 1.55 s to 1.6 s. Before translation, its distance to a cell (indicated by the red arrow, *t* = 1.4 s–1.55 s, denoted as cell C) is *R*_A_ = ~100 *μ*m. If we assume a hypothetical, small bubble (let us denote it as bubble B, radius *R*_B_) adjacent to cell C is about to collapse to cause the cell detachment (shown in the insert in Figure 8), which follows the same physical picture as Figure 7(b) shows; then the bubble wall velocity, *V*_B_, can be extrapolated from the movement of bubble A according to the conservation of mass for an incompressible fluid [24]:

(3) $$\begin{array}{c} V_{B}=V_{A}\cdot \frac{r^{2}}{{R_{B}}^{2}}. \end{array}$$

Here, *V*_A_ = 0.34 cm/s and *r* = 100 *μ*m. The associated fluid speed *V*_*f*_ due to bubble B's collapse can be assumed to be *V*_*f*_ = *V*_B_ at the onset of the bubble collapse. The collapse-induced shear force near cell C can then be estimated as [25]

(4) $$\begin{array}{c} \tau =\mu \cdot \frac{V_{f}}{R_{B}}, \end{array}$$

where *μ* is the dynamic viscosity of the medium (*μ* = 10^−3^ Pa·s). Here, because *R*_B_ is unknown, we assume *R*_B_ to be in the range of 20 *μ*m to 1 *μ*m and so *τ* can be calculated as shown in Figure 8. Smaller bubbles produce larger *τ* and large bubbles (>20 *μ*m) are expected to produce trivial shears. The critical pressure for direct jet impact to induce membrane rupture was reported as 3 kPa [26] and for shear stress to detach a cell is 0.1 kPa [27]. In Figure 8, the estimated shear force sufficient to cause adherent cells to detach is >0.1 kPa for bubble radius < 7 *μ*m. However, in the experiment, a bubble with a radius of 15 *μ*m can detach the cell (Figure 7(a)), but the shear force is only calculated as 10 Pa in Figure 8, an order of magnitude less than the reported threshold (0.1 kPa). This deviation could be due to the oversimplified model we used that precludes the bubble expansion and collapse dynamics and so the actual shear in the experiments can be much larger. But the model at least suggests the possibility of generating sufficient shears for cell detachment with the bubble size observed in our experiments.

It is also possible that other forces may contribute to the cell fragmentation and detachment. When bubbles are emitted from the electrode under intense electric field, the bubble expansion is similar to streamer discharge [28], where a narrow current carrying bubble is partially ionized, resulting in the Joule heating of the bubble, which in turn increases the pressure of the bubble. The expanded bubble acts like a piston on the surrounding water and produces a shock. In an electrode energized by nanosecond pulses for an electric field of 1 MV/cm, the shock pressure was found exceeding 1 GPa in the vicinity of the electrode, but it also decreases rapidly [28]. In addition, the electrostriction resulting from high intensity pulses can contribute to the cell fragmentation and detachment. The electrostriction can be understood that water dipoles will align with the external field and cause the change to its density and pressure. When the electric field energy density is comparable to the external pressure, the pressure change is significant. In studying the pressure transient caused by nanosecond pulses [16], it was found that nanosecond electric fields are capable of generating a high frequency (2.5 MHz), high intensity (>13 kPa) pressure transient for an electric field of 13 kV/cm. The origin of the pressure transient is most likely electrostriction, as the change of the pressure polarity, that is, whether it is rarefaction wave or compression wave, matches the rise and fall of the pulse. In the flat part of the pulse, the pressure wave is diminished. It was concluded, however, that this pressure transient alone was not responsible for membrane poration. Although these forces are viable mechanisms for cell fragmentation and detachment, the principal cause still should be shear force resulting from bubble collapse. A shear force (transverse wave) acts on a cell by twisting it, whereas a compression or a tension force (longitudinal wave) compresses or pulls the cell [29]. Cells exhibit a stronger resistance to compression or tension than shear force.

On the other hand, the cells in the crater periphery (Figure 5) experienced much milder attack and still adhered to the cover slip. Yet, they were permeabilized, as indicated by the PI uptake (Figure 5). The bubble motion and instability could cause microstreaming force to cause permeabilization [30]. In addition, the cell permeabilization can also be caused by pulsed electric fields. The electric field at a distance of ~100 *μ*m from TPE was found to be 90 kV/cm, which is lower than 200 kV/cm, the field required to cause cell permeabilization in single-shot experiments previously reported for 0.5 ns pulses [5, 7]. But the temperature rise and intermittent jet pressure and microstreaming forces may likely assist in the membrane permeabilization. For studying the precise mechanism, these cells in the crater periphery are useful for further analysis to gain more understanding of how ultrashort pulses act on cells, which may ultimately allow us to apply pressure and heat as sensitizing agents to increase the efficiency of ultrashort pulses.

This work has shown the feasibility of generating bubbles by 1 ns pulses to induce cell fragmentation and cell detachment. Certainly, not every bubble collapsed to cause harm to the cells and most bubbles appeared benign. It was reported previously that only inertial cavitation bubbles that contain high pressure water vapor are poised to collapse and cause damage to cells [31, 32].

In our case, the formation of water vapor can be illustrated through a crude calculation near the TPE. The electric field was calculated as 2 MV/cm, and for the conductivity of culture medium (1.5 S/m), the local power density is Δ*P* = 45 GW/cm^3^. Assuming adiabatic conditions (i.e., no heat flow or radiation or chemical losses), which is reasonable for 1 ns pulses, the temperature rise is then approximately Δ*T* = Δ*Pτ*/*ρc*_*p*_, where *τ* is the pulse application time (1 ns, FWHM), *ρ* is the water density in liquid phase (1 g/cm^3^), and *c*_*p*_ is the heat capacity (4.185 J/g °C). Using these values, Δ*T* can be estimated as 14°C. This is just for a single 1 ns pulse. As the pulses were applied at high repetition rate (1 kHz), the temperature could rise much higher and reach the boiling point of water. To measure the temperature change near the electrode by a single pulse is difficult, which requires sufficient temporal and spatial resolution. This, however, may be measured (or indirectly inferred) by the probe beam deflection technique (PBDT), described by Barnes et al. [33]. Nonetheless, in our case, the heating of the bulk liquid near the electrode can be seen from the temperature increase at various distances from the electrode (Figure 4(b)), which is caused by the heat diffusion from the electrode. The divergent flow of heat suggests that vaporization would be highly localized and cannot be expected to occur throughout the region.

The inertial vapor bubble can change its nature to a noninertial, gas filled bubble in its growth by rectified diffusion and bubble coalescence; therefore not every bubble will impose harm to cells. Rectified diffusion involves an unequal mass transfer across the bubble interface in the rarefaction and compression phases of a pressure wave. Considering that a cell medium contains dissolved air and the TPE electrode surface may trap air particles, a bubble has a larger surface area, therefore leading to more gas being diffused into than out of the bubble. It was suggested that the wall of a bubble thins during its expansion, making it easier for gas to diffuse [27]. Alternately, bubble coalescence involves multiple bubbles coming into contact with each other and forming wall partitions. The partitioning walls rupture when they become sufficiently thin, leading to the formation of bigger bubbles [34]. The large bubbles in our experiment mostly dissolved or burst far away from the TPE electrode and did not cause any PI uptake.

## 5. Conclusions

Delivery of 1 ns pulses to a triple-point electrode has allowed us to create a very large field in treating monolayer cells. The electric field causes a multitude of events including bubble generation and temperature increase. This series of physical stimuli, however, produces localized damage to cells, which includes cell fragmentation in a crater and cell permeabilization in the crater periphery. The crater has a diameter of approximately 100 *μ*m. The cell fragmentation is presumed to be caused by bubble collapse and its associated shear force, whereas the permeabilization is caused by bubble movement and microstreaming, although the pulsed electric field with the assistance of temperature may also have contribution. It is unknown whether these permeabilized cells remain viable and so further survival experiments need to be done. The work in this paper shows that ultrashort nanosecond pulses can be used to drive a triple-point electrode for tissue ablation that requires submillimeter precision. The advantages of such a pulsed electrode may enable a longer electrode lifetime and less plasma formation than long pulse driven electrodes, although more studies are needed to verify that.
